# Genomic landscape of nosocomial *Acinetobacter baumannii*: A comprehensive analysis of the resistome, virulome, and mobilome

**DOI:** 10.1038/s41598-025-03246-7

**Published:** 2025-05-25

**Authors:** Sara Pearl, Anand Anbarasu

**Affiliations:** 1 Department of Biotechnology, School of Biosciences and Technology (SBST), Vellore Institute of Technology (VIT), Vellore, 632014 India; 2 Medical and Biological Computing Laboratory, School of Biosciences and Technology (SBST), Vellore Institute of Technology (VIT), Vellore, 632014 India

**Keywords:** Antimicrobial resistance, Virulence, Mobile genetic elements, Pangenome, *A. baumannii*, Genome, Computational biology and bioinformatics, Data mining, Genome informatics, Phylogeny, Antimicrobials, Bacteria

## Abstract

*Acinetobacter baumannii* (*A. baumannii*) is a major multidrug-resistant pathogen, posing serious threats in the healthcare settings. This study provides a comprehensive genomic analysis of nosocomial *A. baumannii* whole-genome sequences retrieved from NCBI Genome database. Multilocus sequence typing and capsule typing were performed to investigate the clonal diversity. The genomes were characterized to identify antimicrobial resistance genes (ARGs), virulence factors, and mobile genetic elements. Further, pangenome analysis was conducted to examine the core and accessory genomes of *A. baumannii*. Our dataset comprised of 609 genomes deposited from diverse geographic regions worldwide between 2004 and 2024. The genomes showed high clonal heterogeneity, with sequence type ST2 being the predominant sequence type. A total of 185 unique ARGs were identified, with majority of them associated with efflux pump and β-lactamase coding genes. Over 25,000 IS elements were detected, with IS4 family being the prevalent type. High abundance of integron-mediated resistance determinants, especially for aminoglycosides and β-lactams, were identified. The open pangenome window due to its larger accessory genome suggested substantial genome plasticity. Our findings highlight *A. baumannii*’s rapid evolution and resistance potential, emphasizing need for alternative therapeutic strategies. Enhanced surveillance, infection control measures, and antimicrobial stewardship are crucial to combat this persistent threat.

**Supplementary Information:**

The online version contains supplementary material available at 10.1038/s41598-025-03246-7.

## Introduction

*Acinetobacter baumannii* (*A. baumannii*) is a Gram-negative, aerobic, pleomorphic, non-motile coccobacillus^1^. The World Health Organization (WHO) has identified and listed the carbapenem-resistant *A. baumannii* (CRAB) among the 2024 list of ‘Critical’ priority bacterial pathogens due to serious threats caused by the pathogen in the clinical settings^2^. This superbug has been categorized among the major pathogens resistant to multiple antibiotics known as ‘ESKAPE’ (*Enterococcus faecium*, *Staphylococcus aureus*, *Klebsiella pneumoniae*, *Acinetobacter baumannii*, *Pseudomonas aeruginosa* and *Enterobacter* spp). It belongs to the *Acinetobacter calcoaceticus/baumannii* (Acb) complex of the *Moraxellaceae* family, and its genome size ranges from 3.4 to 4.2 Mb^3,4^.

*A. baumannii* is a pivotal pathogen in causing nosocomial, community-acquired, and opportunistic infections, and also has been referred to as “Iraqibacter” due to its association with infections in military medical facilities^5^. It is frequently implicated in healthcare-associated infections (HAIs), including bloodstream infections (BSI), urinary tract infections (UTI), pneumonia, meningitis, and wound infections^6^. These pathogens particularly affect chronically ill and immunocompromised individuals, as well as patients with prolonged stays in intensive care units (ICUs). The primary sites of infection and colonization include the bloodstream, respiratory tract, urinary tract, surgical wounds, skin, as well as, pleural fluids, eye, saliva, cerebrospinal fluid (CSF), and peritoneal fluid. *A. baumannii* is largely attributed to its “persist and resist” strategy, which favors it to thrive in various environments and resist antimicrobial treatments.

The burgeoning prevalence of multidrug-resistant (MDR), extensively drug-resistant (XDR), pandrug-resistant (PDR), and carbapenem-resistant (CR) strains poses significant challenges in the effective treatment of *A. baumannii* infections. In a global cohort study conducted comprising of 114 study centers from 47 countries, 96% of *A. baumannii* isolates were reported to harbor acquired carbapenemase genes^7^. A meta-analysis conducted in 2019 to assess the prevalence of multidrug-resistant *A. baumannii* (MDRAB) in hospital-acquired pneumonia (HAP) and ventilator-associated pneumonia (VAP) estimated a high global mortality rate of approximately 43%^8^. Russo et al. reported 62.9% septic shock episodes to be associated with the *A. baumannii*-caused BSI with 98.6% strains accounting for XDR and 1.4% PDR; The 14-day and 30-day mortality rates were 61.2% and 73.6%, respectively^9^. In the recent years, *A. baumannii* has developed resistance to even the last resort antibiotics such as tigecycline and colistin. Particularly, the colistin resistance was observed to be higher in the South-East Asia and Eastern Mediterranean countries than the other regions^10^.

*A. baumannii*’s potential to rapidly alter its genomic structure through the acquisition of resistance markers under antibiotic pressure *via* antimicrobial resistance genes (ARGs), virulence factors (VFs), and mobile genetic elements (MGEs), further complicates therapeutic strategies. This study aims to identify key genomic determinants of resistance and provide a comprehensive understanding of the genetic diversity observed in the nosocomial *A. baumannii* strains.

## Methods

### Genomic data collection and genome assembly QC

Whole-genome sequences (WGS) for this study were retrieved from the Genome database of National Center for Biotechnology Information (NCBI) repository, released between 2004 and October 2024. From the total 32,068 *A. baumannii* genomes deposited, records classified as atypical, large multi-isolate assemblies, or metagenome-assembled genomes (MAGs) were excluded. This initial level of filtering resulted in 10,071 genomes. Further, the genome entries were filtered for ‘Complete’ level of assembly (*n* = 809) to ensure high assembly quality. Metadata, including isolation source, host, host disease, and geographical location, were manually curated. Further to focus on the human-associated isolates, genomes derived from non-human hosts were excluded, resulting in a final dataset of 609 genomes. The quality of genome assemblies was evaluated using QUAST v.5.3.0, while completeness was assessed with BUSCO v.5.8.2^11,12^.

### Gene prediction and functional annotation

Gene prediction and annotation of structural and functional elements including coding sequences (CDS), rRNA, tRNA, tmRNA, miscRNA, and repeat regions was performed using Prokka v.1.14.6^13,14^. Functional annotation based on the cluster of orthologous genes (COGs) was conducted using COGclassifier v.1.0.5.

### Sequence typing and capsule typing

Multilocus sequence typing (MLST) and capsule typing were performed using PathogenWatch v.23.1.6. MLST detection was based on housekeeping genes of two schemes from PubMLST: the Oxford scheme (*gltA*, *recA*, *cpn60*, *gyrB*, *gdhB*, *gpi*, *rpoD*) and the Pasteur scheme (*gltA*, *recA*, *cpn60*, *fusA*, *pyrG*, *rplB*, *rpoB*)^15^. Capsular polysaccharide loci (KL) and outer core lipooligosaccharide loci (OCL) typing was based on the Kaptive database^16,17^.

### Antimicrobial resistant gene and virulence factor identification

Resistance genes were identified using the ABRicate v.1.0.1 against the Comprehensive Antibiotic Resistance Database (CARD)^18,19^. Virulence genes responsible for pathogenicity were identified using the ABRicate search against Virulence Factor Database (VFDB)^20,21^.

### Mobile genetic elements, CRISPR-Cas, and Restriction-Modification system identification

Insertion sequence (IS) elements were predicted using the ISEscan tool v.1.7.2.3^22^. The integron sequences were identified using IntegronFinder v.2.0.5^23–27^. Additionally, the prophage regions were detected using Phigaro v.2.4.0^28^. CRISPR-Cas regions were identified using the machine learning-based tool CRISPRcasIdentifier v.1.1.0^29^. Extremely Randomized Trees (ERT) algorithm was used for the classifier and regressor with hidden Markov model HMM2019. Further, the annotated GBK files of genomes were screened to detect presence of Type-II restriction-modification systems (RMS) using rmsFinder against REBASE database^30,31^. Type-II RMS comprises of restriction endonucleases (REases) and DNA methyltransferases (MTases) enzymes.

### Pangenome analysis

Pangenome analysis was performed using Panaroo v.1.5.1 and Roary v.3.13.0^32,33^. Annotated GFF files from Prokka output were processed with Panaroo for core gene alignment using built-in MAFFT alignment tool. Panaroo outputs included core gene alignment, gene presence-absence matrix, gene network graph. Core genome-based phylogeny was constructed using the IQ-TREE, employing the General Time Reversible (GTR) substitution model^34^. Gene presence matrix clustered based on the phylogeny and pangenome distribution plots were generated using Roary with output tree and gene presence-absence matrix.

## Results

### Genomic dataset retrieval and quality assessment

**Fig. 1:**
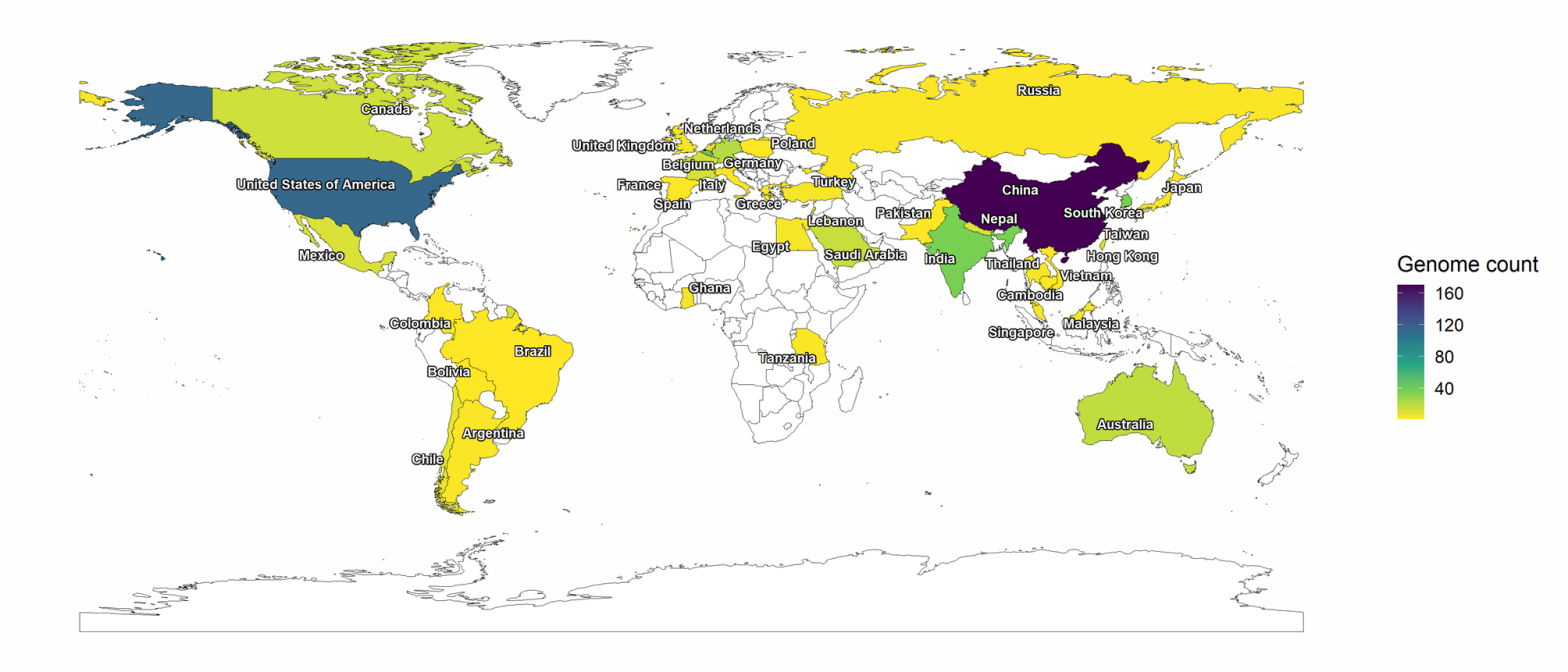
World map illustrating geographic distribution of *A. baumannii* genomes included in this study. Number of genomes contributed by each country is represented with light to dark gradient indicating increasing genome counts. A total of 21 genomes lacked geographic metadata and are categorized as “NA”.

The dataset analyzed in this study comprised of 609 human host-associated *A. baumannii* genomes, primarily of clinical origin. Most of the genomes were derived from isolates causing BSIs, pneumonia and other RTIs. Major isolation sources were sputum and blood, followed by wound samples and other respiratory fluids (**Supplementary Fig. ****S1**). The genomes in our dataset were deposited from various geographical regions, with the majority from China (27.9%) and the United States (18.6%), followed by Belgium (7.2%), South Korea (6.1%), and India (5.9%) (**Supplementary Table ****S1****a**, Fig. 1). Genome sizes ranged from 3.6 to 4.6 Mbp with an average length of 4 Mbp. The average GC content was 39% and the average N50 value was 3.97 Mbp (**Supplementary Table ****S1****b**,** Supplementary Fig. ****S2**). The BUSCO completeness scores of all genomes exceeded 97% with an average of 99.5% (**Supplementary Table ****S1****c**). The assessed quality metrics confirmed the high quality of the dataset.

### Gene prediction and functional annotation

Prokka annotation revealed the predicted gene count, which averaged 3,957 per genome, varying between 3,515 and 4,511. On average, each genome contained 3,826 CDS, with a range spanning from 3,388 to 4,372. Among non-coding elements, the annotation identified an average of 40 miscRNAs, 18 rRNAs, and 73 tRNA per genome (**Supplementary Table ****S2****a**). Functional annotation and categorization using COGclassifier showed an average of 79% of sequences assigned to COG functional groups, with values ranging from 73.1 to 84.8%. The number of classified sequences per genome varied between 2,834 and 3,334, out of a total sequence range of 3,388 to 4,372 (**Supplementary Table ****S2****b** and **S2****c**,** Supplementary Fig. ****S3**). These results highlight the genomic composition and functional diversity of *A. baumannii* isolates.

### Sequence typing and capsule typing

**Fig. 2:**
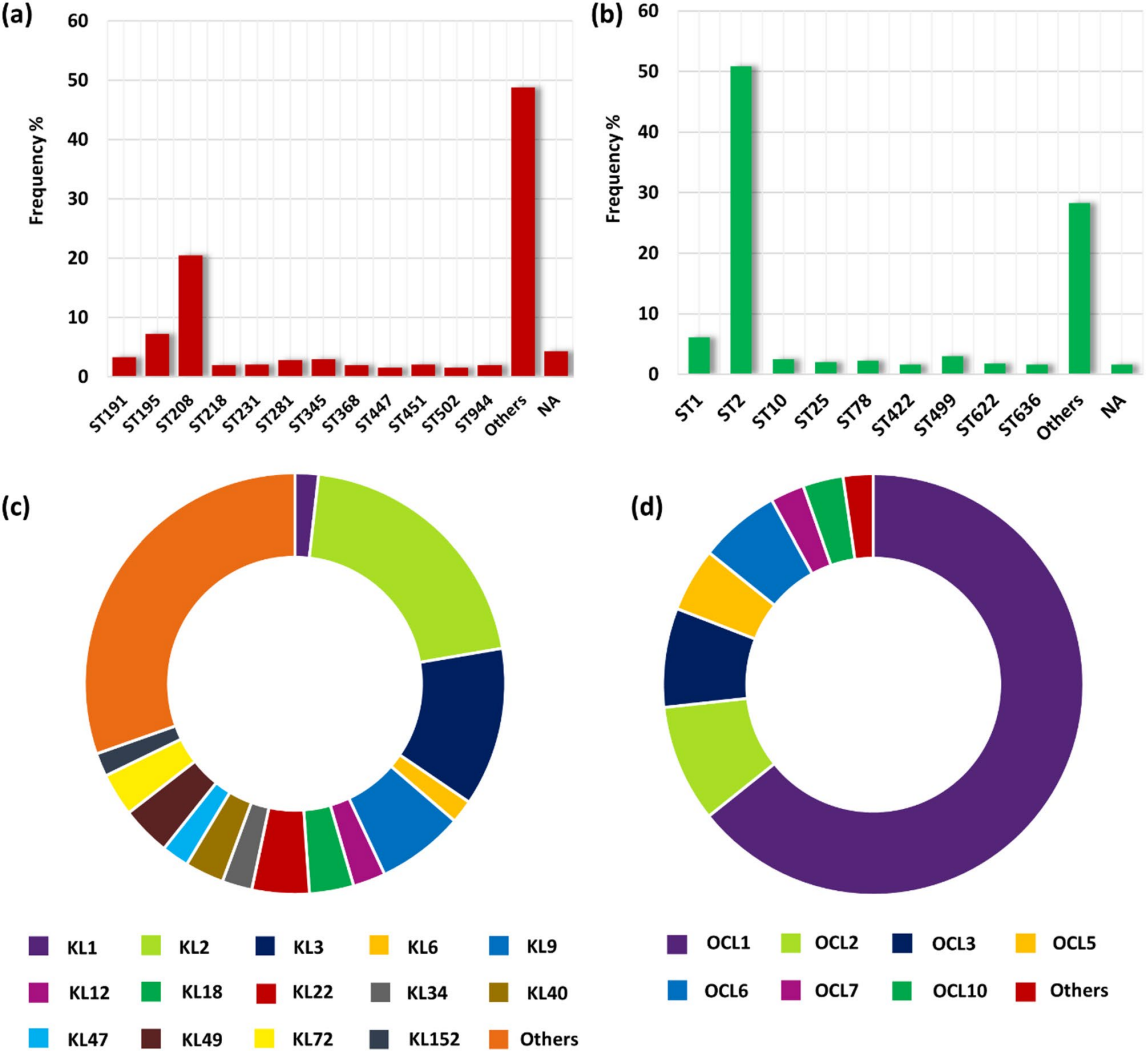
Prevalence of sequence and capsule types in the genomic dataset (**a**) Sequence types according to the Oxford scheme MLST (**b**) Sequence types according to the Pasteur scheme MLST (**c**) K locus (KL) types responsible for capsular polysaccharide synthesis (**d**) Outer core locus (OCL) types responsible for lipooligosaccharide outer core synthesis.

MLST analysis using the Oxford scheme identified 168 sequence types and ST208 was observed to be the dominant sequence type (20.5%) in the dataset, followed by ST195 (7.2%). The Pasteur MLST scheme identified 96 sequence types and ST2 was the most prevalent type (50.9%) (Fig. 2a and b). These findings show the clonal diversity among the *A. baumannii* strains. Capsule typing identified 88 distinct KL types. The most frequently occurring types were KL2 (*n* = 125, 20.5%) and KL3 (*n* = 74, 12.2%), followed by KL9, KL22, and KL49. The total OCL types identified is 14, with OCL1 (*n* = 391, 64.2%), OCL2 (*n* = 55, 9%), and OCL3 (*n* = 46, 7.6%) being the most prevalent. Other frequently occurring locus includes OCL6 and OCL5 (Fig. 2c and d). Detailed sequence type and capsule type distribution are provided in the **Supplementary Table ****S3**.

### Resistome and virulome profiling

**Fig. 3:**
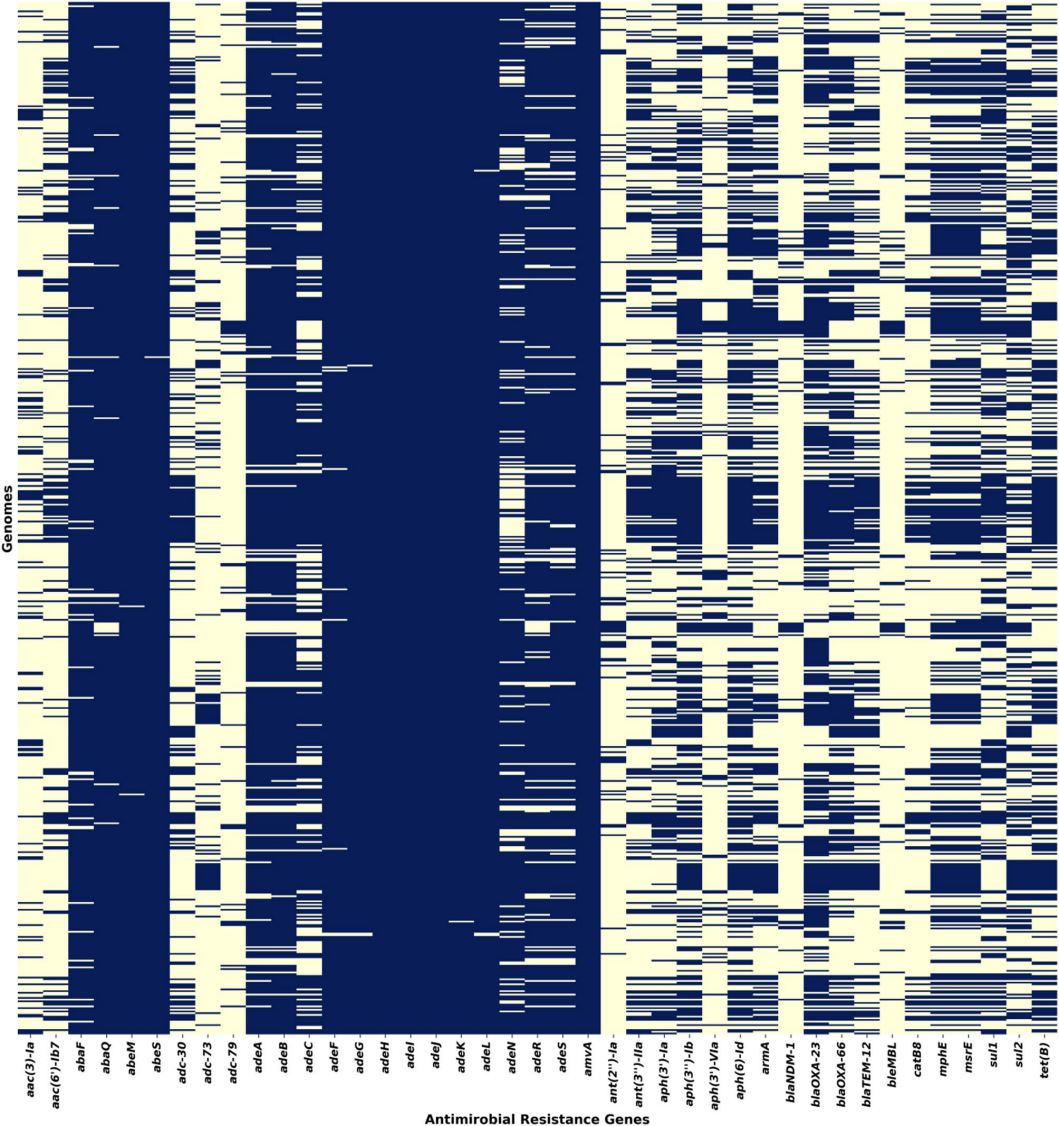
Heatmap showing the presence of prevalent ARGs in the dataset, with presence indicated in blue.

A total of 185 unique ARGs were identified across the dataset (**Supplementary Table ****S4**). A heatmap for the 41 frequently occurring ARGs (occurring in > 50 genomes) is presented in Fig. 3. Notably, *amvA*, *adeH*, *adeI*, and *adeJ* genes were present in all genomes, while *abeS*, *adeK*, *abeM*, *adeG*, *adeL*, *adeF*, *abaQ*, *abaF*, and *adeB* were detected in at least 90% of the dataset (**Supplementary Fig. ****S4**). These genes codes for the efflux pump systems, majorly conferring resistance to fluoroquinolones and tetracyclines.

ARGs were classified based on drug resistance mechanisms. The highest number of ARGs were found to confer resistance to cephalosporins (107 ARGs) and penam antibiotics (82 ARGs). Specifically, genes encoding β-lactamase enzymes such as ADC, CTX-M, GES, GIM, IMP, NDM, OXA, PER, SHV, TEM, TMB and efflux pump genes (*adeI*, *adeJ*, *adeK*, and *adeN*) has primary attribution to the cephalosporin resistance.

**Fig. 4:**
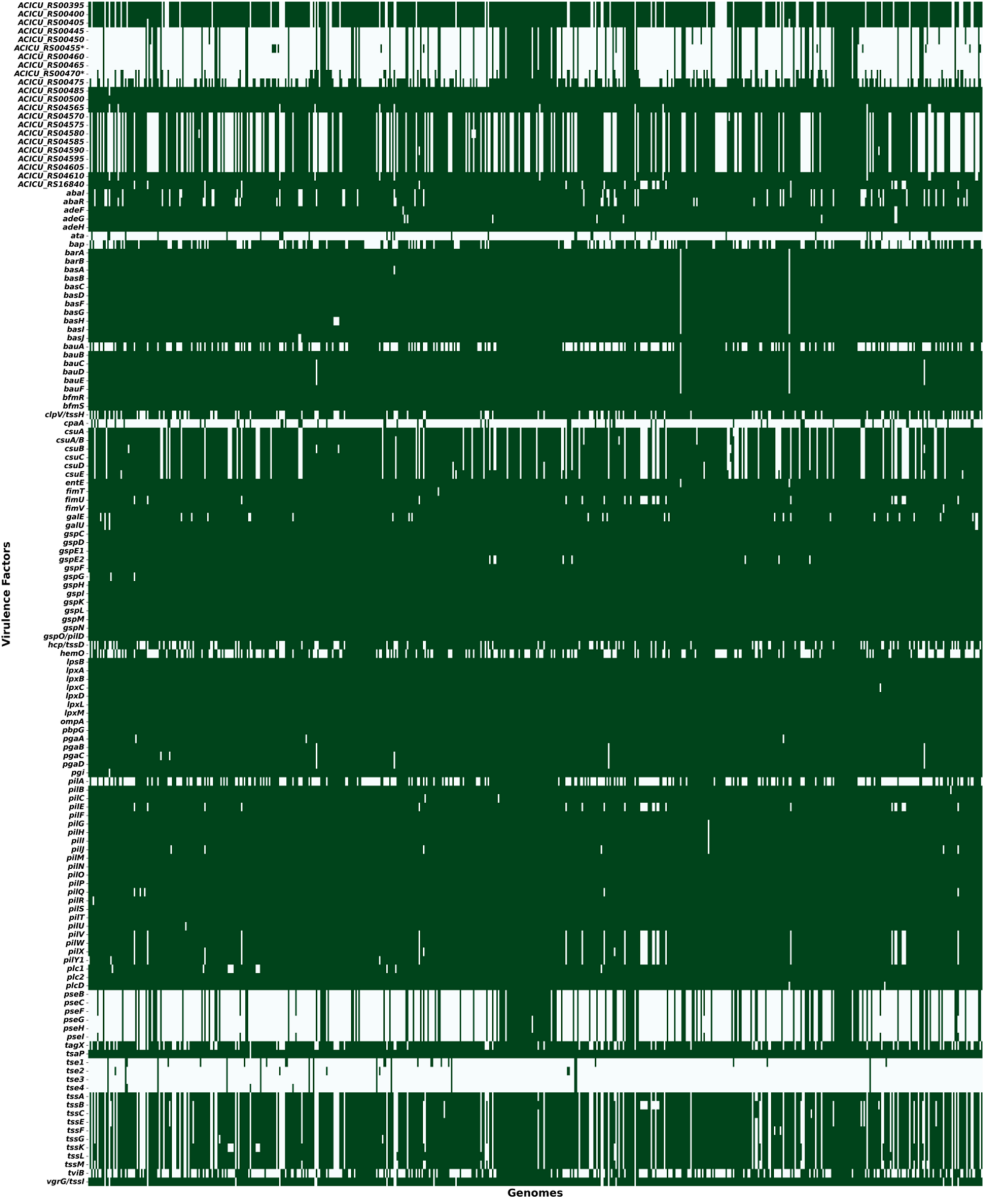
Heatmap representing the distribution of VFs in the dataset, with presence indicated in green.

VF identification revealed 139 unique virulence genes and the most frequently occurring genes were responsible for virulence mechanisms such as immune modulation, adherence, effector delivery systems, and nutritional/metabolic factors. A core set of 31 virulence genes was found to be present across all genomes, highlighting key factors contributing to *A. baumannii* pathogenicity. A heatmap illustrating the distribution of VFs is shown in Fig. 4.

### Mobilome profiling

**Fig. 5:**
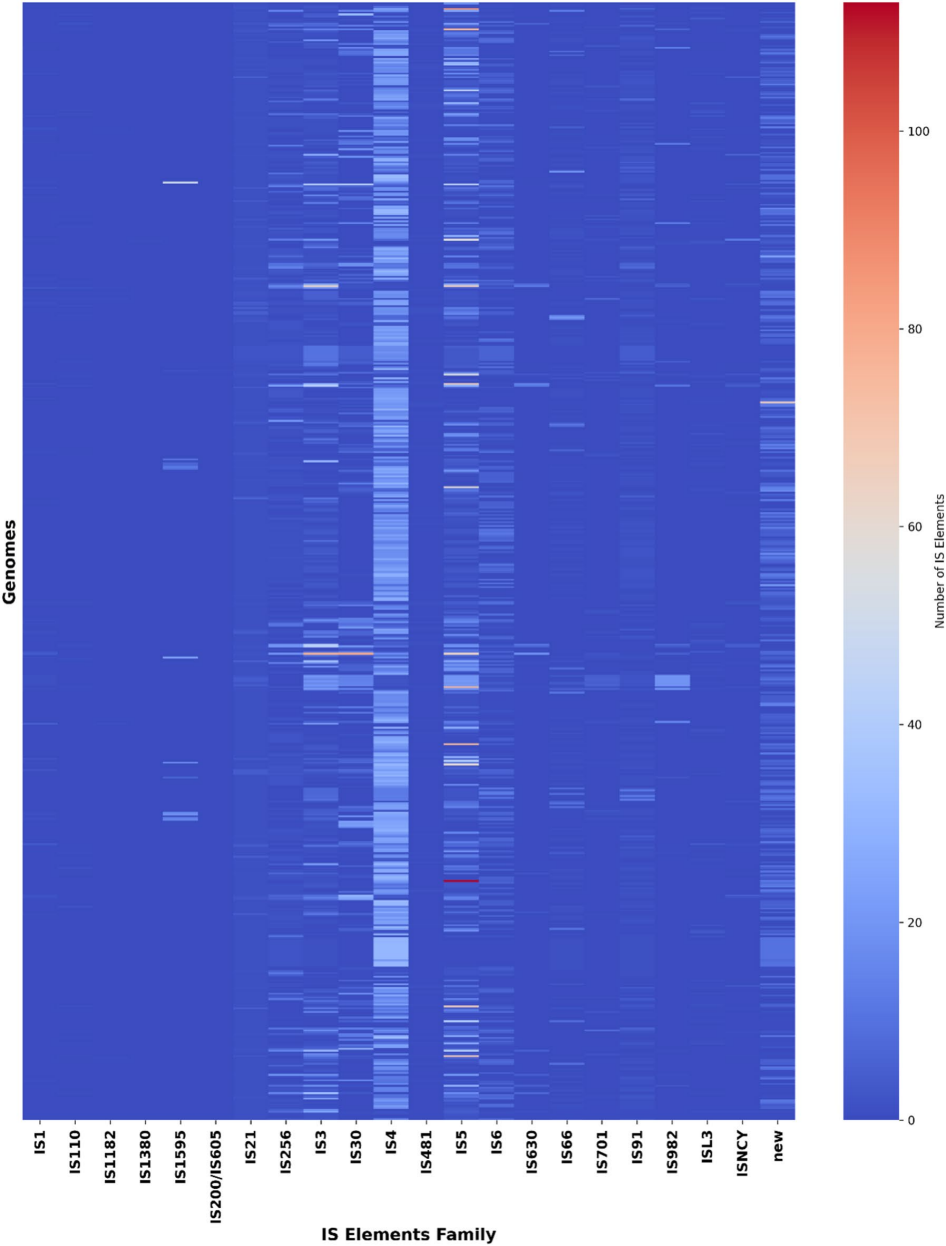
Heatmap depicting the distribution of IS elements across the dataset.

A total number of 25,127 IS elements were identified across all 609 genomes, occupying 1.4% of the genome on average. GCA_024749505.1 genome had an extremely higher number of insertions (297 IS), covering 8.87% of the whole genome. It is observed that 125 genomes (20.5%) harbored more than 50 IS elements per genome. A total of 21 IS families were detected, with the most prevalent IS families being IS4, IS5, IS21, and IS3, followed by IS256 and IS91. Additionally, a set of unclassified IS elements (denoted as “new”) was identified, with a frequency of 2328 sequences across the dataset (Fig. 5, **Supplementary Table ****S5****a**,** Supplementary Fig. ****S5**).

**Fig. 6:**
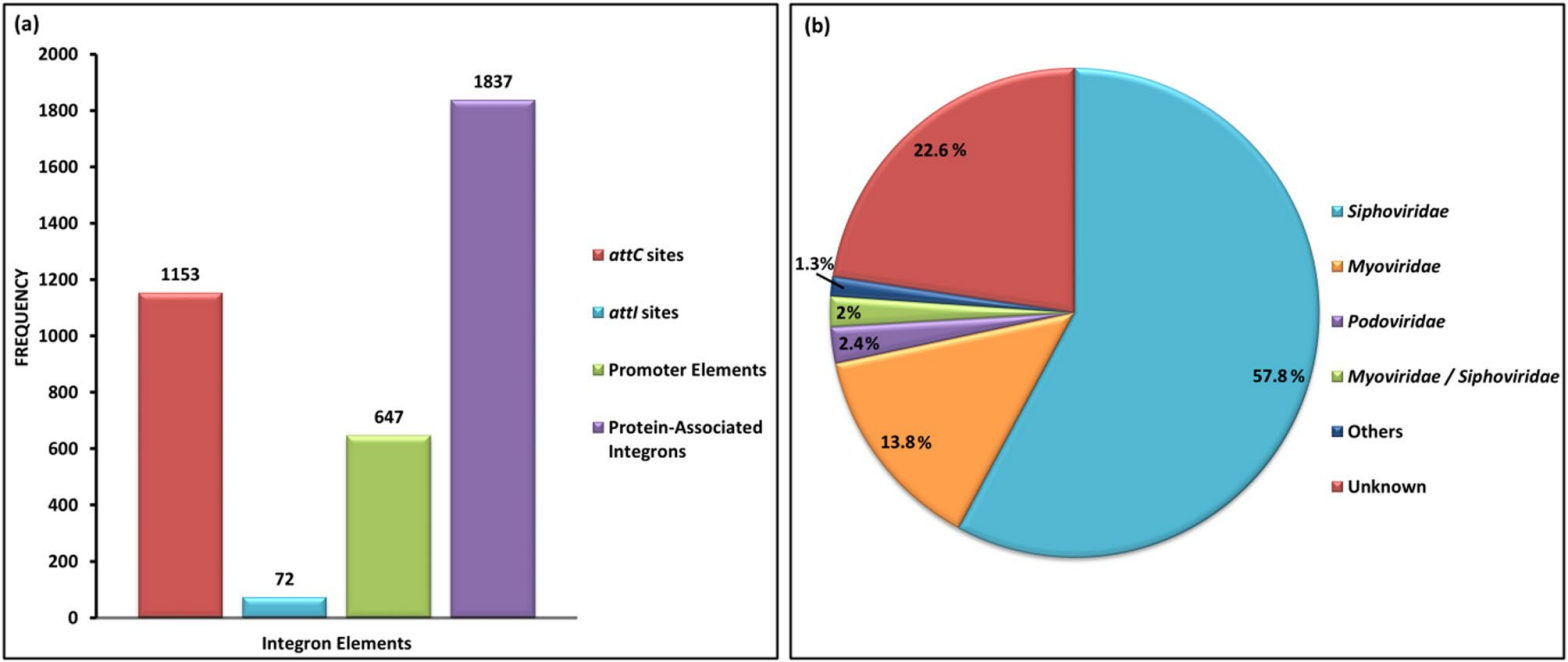
Plots illustrating distribution of integrons and prophages occurring in the dataset (**a**) Frequency of integron-associated elements (**b**) Prevalence of taxonomic lineages of the prophage regions.

A total of 3,709 integrons were detected in most genomes (*n* = 313), indicating a high prevalence of integron-mediated genetic elements and variability in horizontal gene transfer (HGT) mechanisms across different isolates. The most prevalent integron elements responsible for gene cassettes integration are the *attC* sites (frequency = 1,153). The protein-associated integron elements (frequency = 1,837) comprised majorly of the integrons associated with antibiotic resistance. The key integron-mediated resistance determinants included genes conferring resistance to aminoglycosides [$aac(6')-Ib$ and $ant(3'')aadA1$] and β-lactams (*bla*_*OXA−2−like*_, *bla*_*IMP*_, and *bla*_*GES*_). Integron-associated regulatory elements included *intI* integrase, along with the promoters, P_c1_ and P_int1_ (Table 1; Fig. 6a, **Supplementary Table ****S5****b**).

**Table 1:** Distribution and frequency of integron-associated elements.

Integron element type	Function	Integron	Frequency
Recombination sites	Gene cassettes integration	*attC*	1153
		*attI1*	72
Regulatory elements	Integrase - gene cassette mobility	*intI*	347
	Promoter - transcriptional activation	P_c1_	315
		P_int1_	332
Antibiotic resistance	Aminoglycoside resistance	*aac(3)-I*	115
		$aac(6')-Ia$ family	6
		$aac(66')-Ib$	171
		$ant(2'')-Ia$	47
		$ant(3'')aadA1$	258
		$ant(3'')-I$	55
		$aph(3')-I$	1
	Streptothricin resistance	*sat2*	20
	β-lactam resistance	class D β -lactamase	2
		*bla*_*GES*_	10
		*bla*_*GIM*_	1
		*bla*_*IMP*_	1
		*bla*_*OXA−2−like*_	8
		*bla*_*TMB*_	1
		*pse*	8
	Chloramphenicol resistance	*catB*	152
		*cmlA*-*floR*	2
		*cmlA*	33
	Rifampin resistance	*arr*	34
	Efflux-mediated disinfectant resistance	*qacE* (SMR family)	323
	Sulfonamide resistance	*sul1*	2
	Trimethoprim resistance	*dfr*A1*-like*	32
		*dfr*A12	1
		*dfr*B	1
Other protein-associated integron elements	-	Other proteins	206

Prophage region analysis revealed a total of 2,989 prophage regions and they were classified into specific viral lineages based on their homology to known virus orthologous groups (VOGs). Majority of the identified prophages in the dataset belonged to *Siphoviridae* family (57.85%), followed by *Myoviridae* family (13.75%) (Fig. 6b, **Supplementary Table ****S5****c**).

### Defense system prediction

CRISPR-Cas system of multiple Cas subtypes were confirmed in 49.4% of genomes (*n* = 301), with a total of 438 CRISPR-Cas loci. Both Class 1 (CAS-I-F) and Class 2 systems (CAS-II-C, CAS-V-A, CAS-V-F, CAS-VI-B) were found. The most predominant subtype was CAS-VI-B (*n* = 268), followed by CAS-I-F (*n* = 90) and CAS-V-A (*n* = 77). Less frequent subtypes included CAS-II-C (*n* = 2) and CAS-V-F (*n* = 1).

MTases with experimentally characterized biochemical functions were identified in 606 genomes, ranging from 1 to 4 per genome (average: 1.5). Non-putative MTases were found in 602 genomes, with a range of 1 to 7 per genome (average: 1.8). Predicted MTases (putative) were identified in 606 genomes, ranging from 3 to 13 per genome (average: 7.0) (**Supplementary Table ****S6****a**). REases with experimentally validated function were found in only a single genome (GCA_014672735.1). Notably, this genome comprised both MTase and REase enzymes, and thus was the only genome in the dataset with a complete RMS. Putative REases were found in 429 genomes, with 1 to 3 REases per genome (average: 1.2) (**Supplementary Table ****S6****b**).

### Analysis of pangenome

**Fig. 7:**
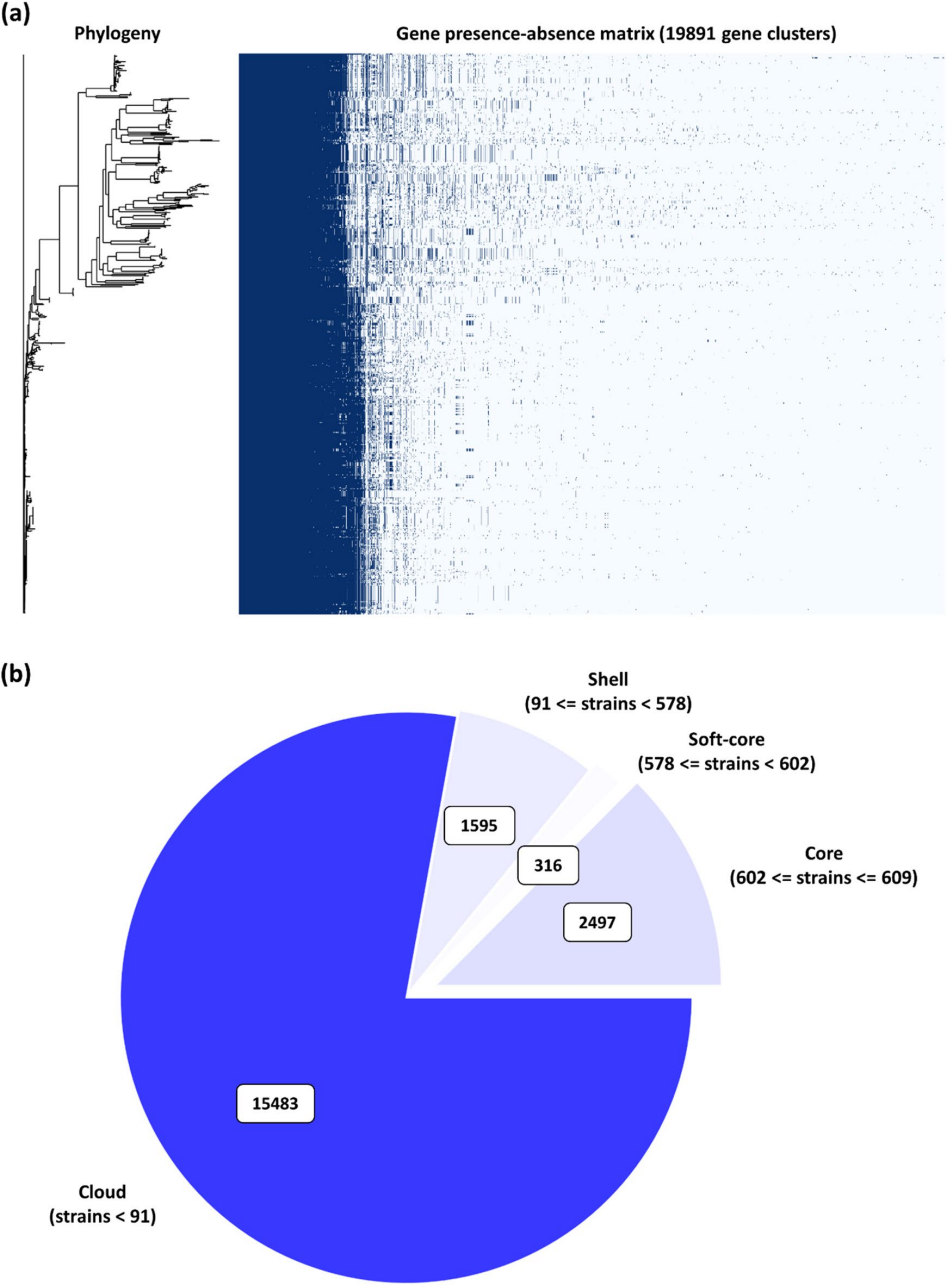
Pangenome of *A. baumannii* (**a**) Core genome-based phylogeny compared against presence-absence matrix of core and accessory genes (**b**) Depicts the proportion of core and accessory genes across the genomes.

The preliminary quality control revealed the number of genes in each genome (**Supplementary Fig. ****S6**). Pangenome analysis revealed 19,891 total number of genes across the genomes in the dataset. The classification of genes revealed 2,497 core genes, 316 soft core genes, 1,595 shell genes, and 15,483 cloud genes. These results provide insight into the genomic diversity and evolutionary trends among *A. baumannii* isolates (Fig. 7a and b).

### Global trends in clonal diversity and genomic features

Upon comparing the sequence type profiles (Pasteur scheme) of the genomes across the geographical regions, ST2 was observed to be the dominant type in Asia (65.2%), the Americas (39.3%), and Europe (32.6%). In the Americas, ST499 emerged as the second most prevalent sequence type (11.3%). Notably, this ST499 was not detected in genomes from any other region, suggesting possible regional adaptation or limited dissemination. In Europe, ST1 (16.8%) and ST636 (10.5%) were identified as the second and third most prevalent types, respectively. Similar to ST499, ST636 was uniquely found only in the European genomes and was absent in other region genomes of the dataset (**Supplementary Fig. ****S7**).

The variations in prevalence patterns of KL types were observed across different regions. In Asia, KL2 (24.1%) was observed to be highly prevalent KL type followed by KL3 (8.5%), KL72 (6.0%), and KL49 (5.4%). Similarly, in the Americas, KL2 (22.7%) remained the dominant KL type where as KL18 (12.7%), KL22 (10.0%), and KL9 (10.0%) showed significant prevalence. In contrast, KL40 (16.8%) was observed to be the most prevalent KL type, followed by KL3 (13.7%), KL9 (10.5%), and KL2 (9.5%) in Europe. The outer core type OCL1 was observed to be highly prevalent in Asia (78.2%), the Americas (54%), and Europe (48.4%). The dominance of OCL1 was followed by OCL3 (20.7%) in the Americas and OCL2 (20.0%) in Europe.

A summary of region-wise distributions of ARGs, VFs, IS elements, prophages is provided in **Supplementary Table ****S7****a**. On average, each genome harbored approximately 27 ARGs, with highest regional average of Asia (28.3), followed by Europe (25.7) and Americas (25.6). The average number of VFs per genome was 116.6 in Asia, 114.5 in the Americas, and 114.2 in Europe. Comparison of high IS element abundance (> 50 IS elements per genome) revealed a higher proportion in genomes from the Americas (26.7%; 40/150) and Europe (30.5%; 29/95) than those from Asia (14.2%; 45/316). The average number of prophage sequences per genome was relatively uniform across regions, ranging from 3.0 to 3.6.

### Source-specific comparison of genomic features

The comparison of genomes based on isolation sources revealed variations in clonal lineages and genomic features (**Supplementary Fig. ****S1**). The sequence type ST2 (Pasteur scheme) emerged as the most dominant type across the major isolation sources, with the prevalence rate of 65% in sputum-derived genomes, 60% in blood, and 44% in wound. Moreover, KL2 was the most frequently identified KL type in sputum (*n* = 41), blood (*n* = 25), and wound (*n* = 12) samples.

Source-wise distribution summary of ARGs, VFs, IS elements, and prophages is tabulated as **Supplementary Table ****S7****b**. ARG comparison profiles revealed that *aac(6’)-Ib7* and *ant(3’’)-IIa*, both conferring resistance to aminoglycosides along with *bla*_*ADC−30*_ associated with cephalosporin resistance were enriched in genomes of blood samples. In contrast, *bla*_*TEM−12*_ conferring resistance to cephalosporins, monobactams, penams, and penems was observed to be more prevalent in genomes of sputum samples. Notably, Type VI Secretion System (T6SS) gene cluster (*clpV*/*tssH*, *hcp*/*tssD*, *tagX*, *tssA*, *tssB*, *tssC*, *tssE*, *tssF*, *tssG*, *tssK*, *tssL*, *tssM*) responsible for virulence *via* effector delivery, was found to be enriched in the genomes of wound samples.

## Discussion

*A. baumannii* as a key pathogen in nosocomial infections has gained clinical significance due to its rapid surge in MDR, leading to increased morbidity and mortality. It harbors numerous genomic islands rich in resistance genes and MGEs, enabling it to switch its genomic structure. The study revealed significant clonal diversity in *A. baumannii* strains, with ST2 (Pasteur scheme) being the most prevalent sequence type. ST2 has been globally associated with CRAB outbreaks, highlighting its epidemiological importance^35–37^. Our findings from global comparison of sequence type profiles aligns with an MLST epidemiological correlation study’s conclusion of regional variability in the clonal diversity of *A. baumannii*^38^. This highlights the significance of incorporating geographic context into epidemiological and genomic surveillance. Moreover, ST2 emerged as cross-tissue prevalent clone across most sample types, suggesting hypervirulence potential of ST2^39^. Particularly in the nosocomial settings, the high degree of variation in the capsule types in *A. baumannii* strains aids in immune modulation and resistance to desiccation and complement-mediated killing^40^. Additionally, it helps in adhesion to host cells, hospital surfaces and instruments, further increasing the virulence levels of the pathogen^41^. KL2 was consistently co-associated with OCL1, indicating the widespread dominance of possible virulent strains.

The detection of 185 unique ARGs constituting the *A. baumannii*’s resistome emphasizes heterogeneity in its intrinsic and acquired resistance mechanisms. Efflux pump system-mediated resistance to fluoroquinolones and tetracyclines remains a major challenge in treating infections caused by this pathogen^42^. Although efflux pump genes are highly prevalent throughout the dataset, the genes conferring resistance to β-lactams (cephalosporins and penams) exhibit significant diversity. This diverseness of genes coding for β-lactamases (OXA, NDM, IMP, ADC, GES, and CTX-M families) suggests a high frequency of carbapenemase-producing *A. baumannii* (CPAB) which is an established global health threat^43,44^. Virulome profiling revealed a core set of 31 VFs which underscores the conserved pathogenic potential of *A. baumannii*. The high prevalence of virulence genes involved in immune modulation, adherence, and effector delivery systems suggests an enhanced ability to colonize host tissues and evade host immune responses^41^. These virulence genes augment the pathogen’s survival mechanisms of biofilm formation, antimicrobial tolerance, and immune evasion contributing to chronic infections^45^.

The findings from source-specific comparison suggest that *A. baumannii* adapts to various niches in the clinical settings by acquiring specific genes. Abundance of several ARGs in blood isolates derived genomes may reflect adaptation for traits promoting bacterial survival in systemic infections, while genomes from sputum and wound isolates harbor genetic determinants that aid surface colonization or biofilm formation^46^. One of the major virulence mechanism in *A. baumannii* is the T6SS which acts as a bacterial nanomachine that inject effector proteins and toxins into target cells, aiding the pathogen to colonize and infect the host cells^47^. The predominance of T6SS-associated genes in the genomes derived from wound samples suggests its contribution in biofilm formation and colonization in skin surfaces and open wounds^48^.

The extensive presence of IS elements, integrons, and prophage fragments highlights the critical role of horizontal gene transfer (HGT) in shaping the *A. baumannii*’s genome. IS elements occupying an average of 1.4% of the genome in current dataset suggests a gradual expansion of insertions compared to the previous observations, potentially contributing to rapid dissemination of resistance genes and mobilization of resistance islands^49^. Predominance of IS4 family in the examined dataset al.igns with the previous reports of the widespread prevalence of IS*Aba1* (a member of IS4 family) insertion element in *A. baumannii* strains, suggesting active genome plasticity mechanism in *A. baumannii*. IS*Aba1* is found upstream of *bla*_*OXA−23*_, *bla*_*OXA−51*_, *bla*_*OXA−58*_, *ampC* and upregulates the expression of these genes^50,51^. IS5 family consisting of IS*Aba10*, IS*Aba13*, IS*Aba27* upregulates the expression of *carO* and *adeN* genes^52^.

The high variability in integron-mediated resistance genes across isolates suggests differential exposure to selective pressures, such as antibiotic use in different clinical settings. *A. baumannii* carries class I and II integron gene cassettes associated mostly with resistance to aminoglycosides, β -lactams, and trimethoprims^53^. The identification of key aminoglycoside-modifying enzymes and β-lactamases associated with integrons highlights its direct role in mobilization of these resistance determinants, accelerating the spread of multidrug resistance particularly among clinical isolates. Notably, the integron-mediated *qacE* gene was observed to be present in more than 50% of dataset. The *qacE* gene codes for the protein belonging to small multidrug resistance (SMR) family of transporters and is mostly found on integrons and plasmids which allows rapid dissemination of these genes^54^. The QacE protein confers resistance to antiseptics and disinfectants, raising concerns about the effectiveness of hospital infection control measures in maintaining the sterility of medical equipment^55^. Identification of *Siphoviridae* as the predominant prophage family in the studied dataset corroborate the study conducted by Kumkar et al.^56^. The CRISPR-Cas system prediction revealed that CAS-I-F was the only Class 1 subtype, while all other identified subtypes belonged to Class 2. This indicates that Class 2 CRISPR-Cas systems are prevalent in *A. baumannii*, particularly CAS-VI-B. The prevalence of CAS-I-F in several genomes suggests that some *A. baumannii* strains retain Class 1 systems^57^. The genome (GCA_014672735.1) encoding the complete RMS also harbored a CRISPR-Cas system of CAS-VI-B subtype, along with prophage sequences from both *Siphoviridae* and *Myoviridae* lineages. The co-occurrence of RMS, CRISPR-Cas, and prophages in single genome signifies the potential for complex immune defense system.

The pangenome of *A. baumannii* revealed a highly diverse genetic repertoire, with 2,497 core genes and a large number of accessory (1,595) and cloud genes (15,483). The large accessory genome highlights the presence of open pangenome window in *A. baumannii*. This aligns with previous studies, suggesting extensive genetic variability and potential for adaptation^37,58,59^. This genomic plasticity is crucial for the survival of *A. baumannii* in various environmental niches, including hospital settings where selection pressure is high due to antibiotic exposure. The open pangenome supports the hypothesis of *A. baumannii*’s rapid evolutionary diversification^60^.

The findings of this study highlights the clinical significance of MDRAB. In addition to high prevalence of carbapenem resistance genes, the occurrence of multiple efflux pump systems and virulence factors, underscores the challenge in treating MDRAB infections. Furthermore, presence of genetic determinants conferring resistance to antiseptics and disinfectants complicates infection control measures in hospitals, suggesting re-evaluation of disinfection protocols. The identification of diverse MGEs indicates the potential for rapid dissemination of AMR, emphasizing enhanced surveillance strategies and strict antibiotic stewardship programs to reduce the spread of MDR strains^61^.

While this study provides insights on the genomic landscape of *A. baumannii* strains associated with clinical settings, there are certain limitations. The lack of phenotypic information limits the ability to correlate genotypic predictions with antimicrobial resistance (AMR) phenotypes, virulence expression, and clinical outcomes. The ARGs or VFs present in the genomic sequences are not always expressed. This may lead to overestimation or underestimation of their clinical significance, thereby restricting validation of findings and understanding of genotype-phenotype associations. Future research should focus on longitudinal surveillance and advancements in genomic analysis of *A. baumannii* isolates.

## Conclusion

This study provides an overall picture of the genomic landscape of nosocomial *A. baumannii*, highlighting genetic diversity of its resistome, virulome, and mobilome. The results reveal high prevalence of carbapenem resistance and efflux pump genes, extensive MGEs, and diverse clonal lineages. The wide range of VFs involved in immune evasion, biofilm formation, and antibiotic resistance underscores the clinical challenges. Majority of the genomes analyzed were originated from Asia, the Americas, and Europe. Distinct clonal types and genomic profiles observed across these regions highlight potential regional selective pressures. The emphasis on the source-specific trends in resistance, virulence, and HGT, suggests complex evolutionary dynamics. The open pangenome window due to its large accessory genome suggests the crucial role of HGT in accelerated adaptations of this pathogen. Consequently, this study highlights the urgent need for enhanced genomic surveillance, effective infection control measures, and antimicrobial stewardship to minimize the spread of MDRAB. The extensive genomic heterogeneity and plasticity observed in these isolates leading to increasing levels of AMR calls for the development of novel antimicrobial agents with enhanced antimicrobial activity.

## Electronic supplementary material

Below is the link to the electronic supplementary material.

Supplementary Material 1

Supplementary Material 2

Supplementary Material 3

Supplementary Material 4

Supplementary Material 5

Supplementary Material 6

Supplementary Material 7

Supplementary Material 8

## Data Availability

The genomes dataset generated and analyzed during this study are retrieved from the Genome database of NCBI repository, (https://www.ncbi.nlm.nih.gov/). All data generated or analyzed during this study are included in this published article (and its Supplementary Information files).
