# Supplementary material for: Genomic landscape of nosocomial Acinetobacter baumannii: A comprehensive analysis of the resistome, virulome, and mobilome
Source: Sci Rep. 2025 May 25;15:18203. doi: 10.1038/s41598-025-03246-7 (PMC12104467; doi:10.1038/s41598-025-03246-7)
Supplement: Supplementary file 1 — Supplementary Material 1 [file 41598_2025_3246_MOESM1_ESM.docx]

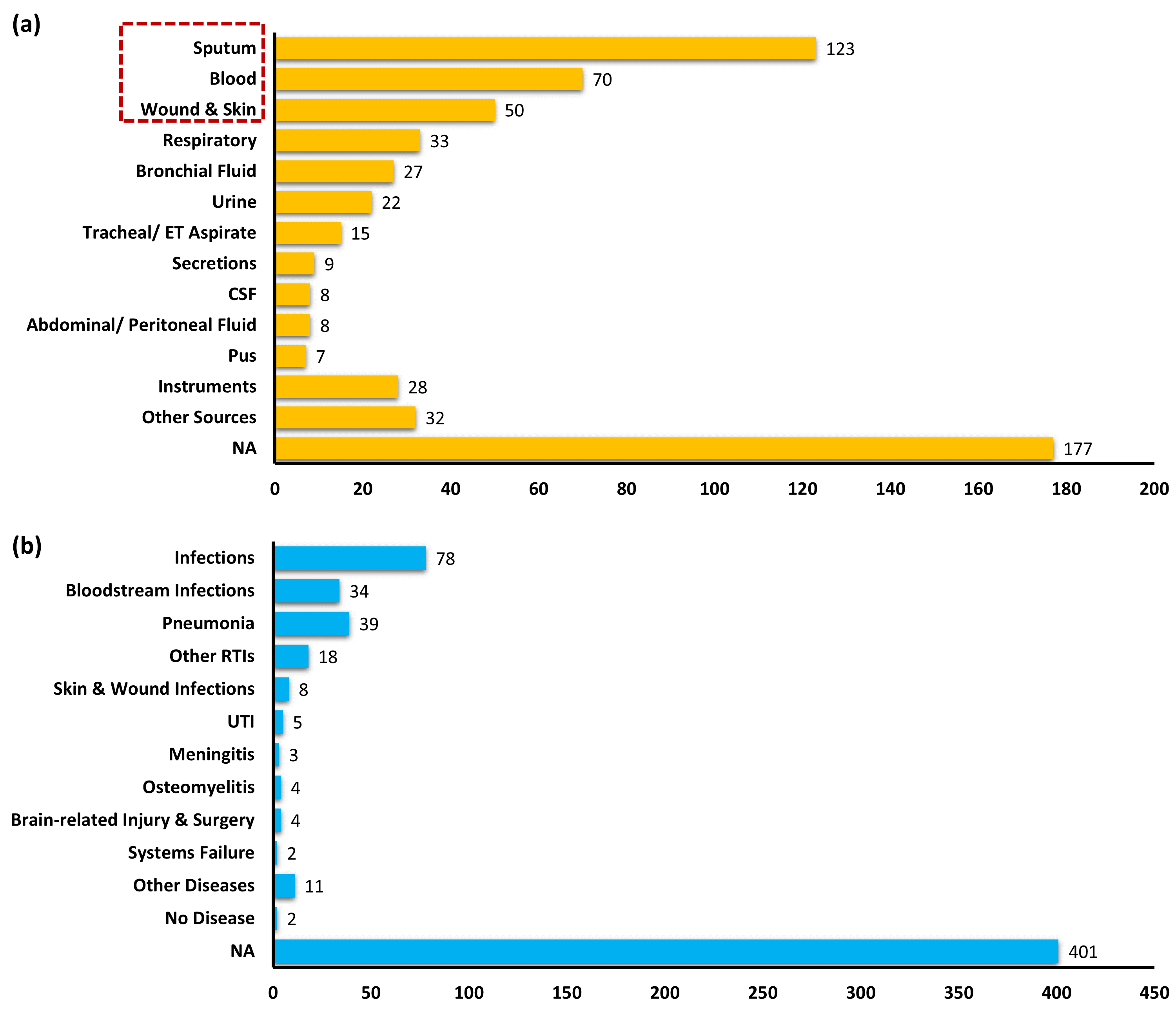


**Supplementary Fig. S1:** Metadata of *A. baumannii* genomes in the dataset (a) Distribution by isolation sources (b) Distribution by associated diseases


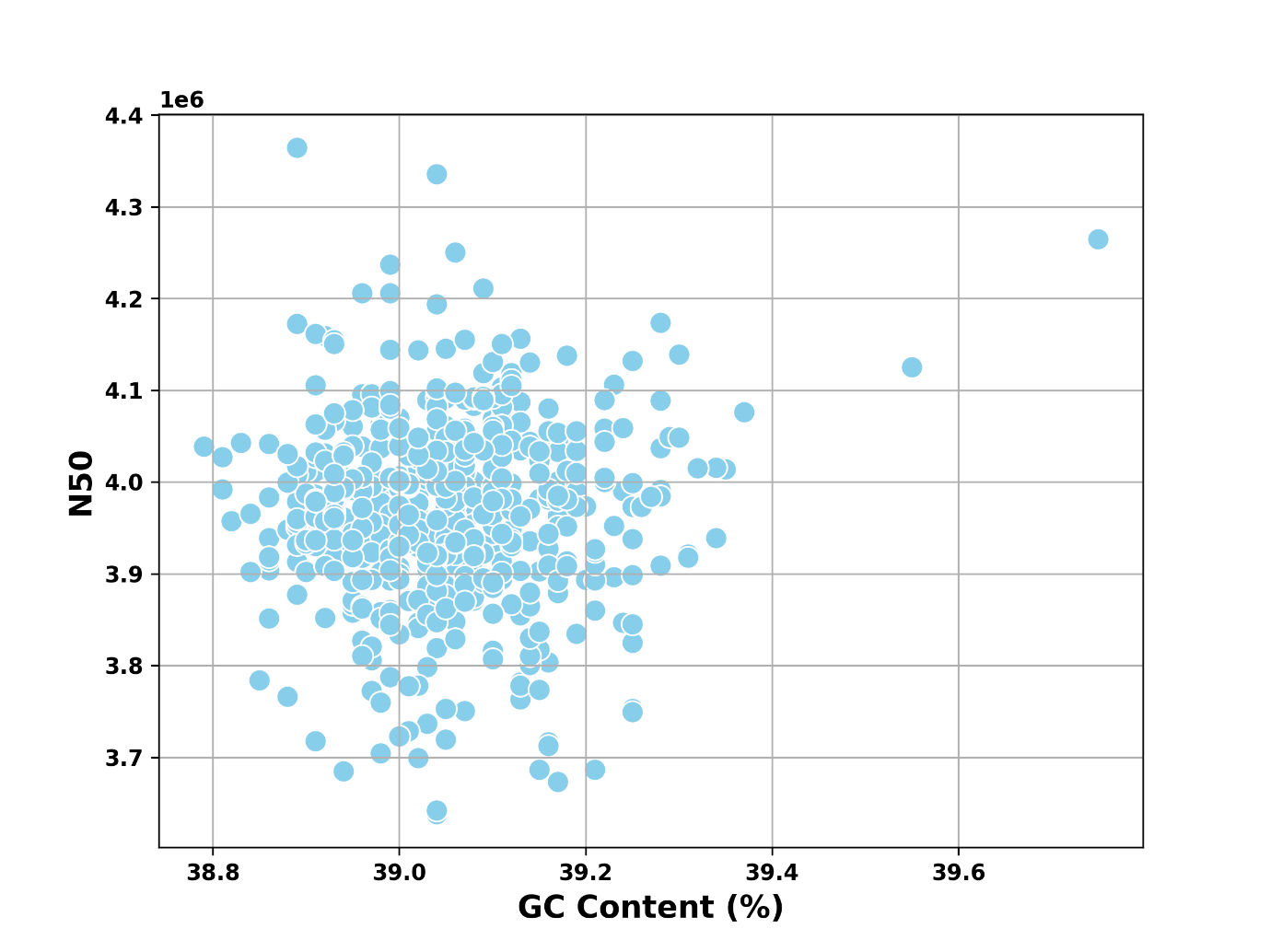


**Supplementary Fig. S2:** Scatter plot illustrating the relationship between GC content (%) and N50 values of genome assemblies in the dataset


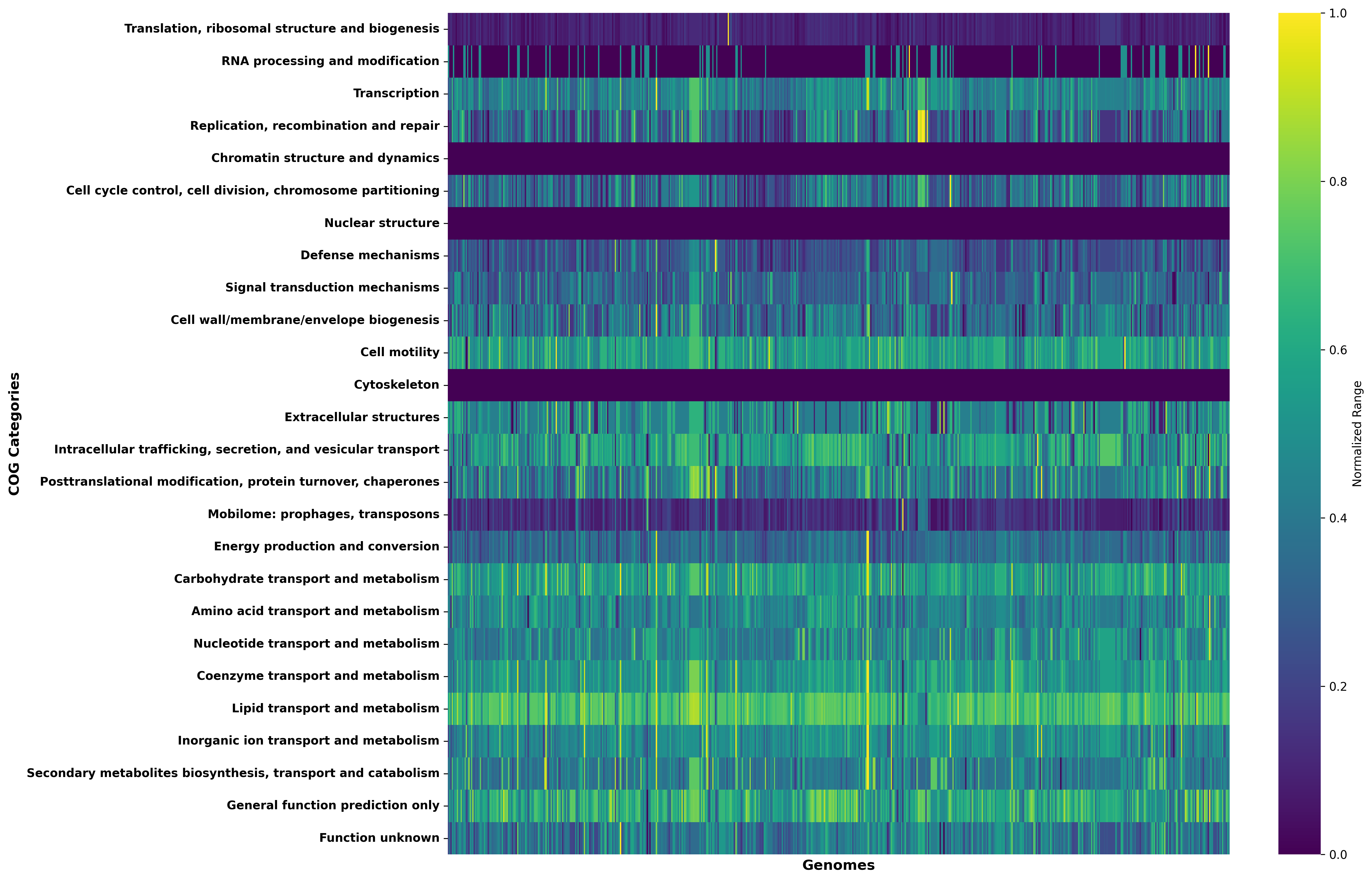


**Supplementary Fig. S3:** Heatmap showing the distribution of cluster of orthologous genes across the functional categories


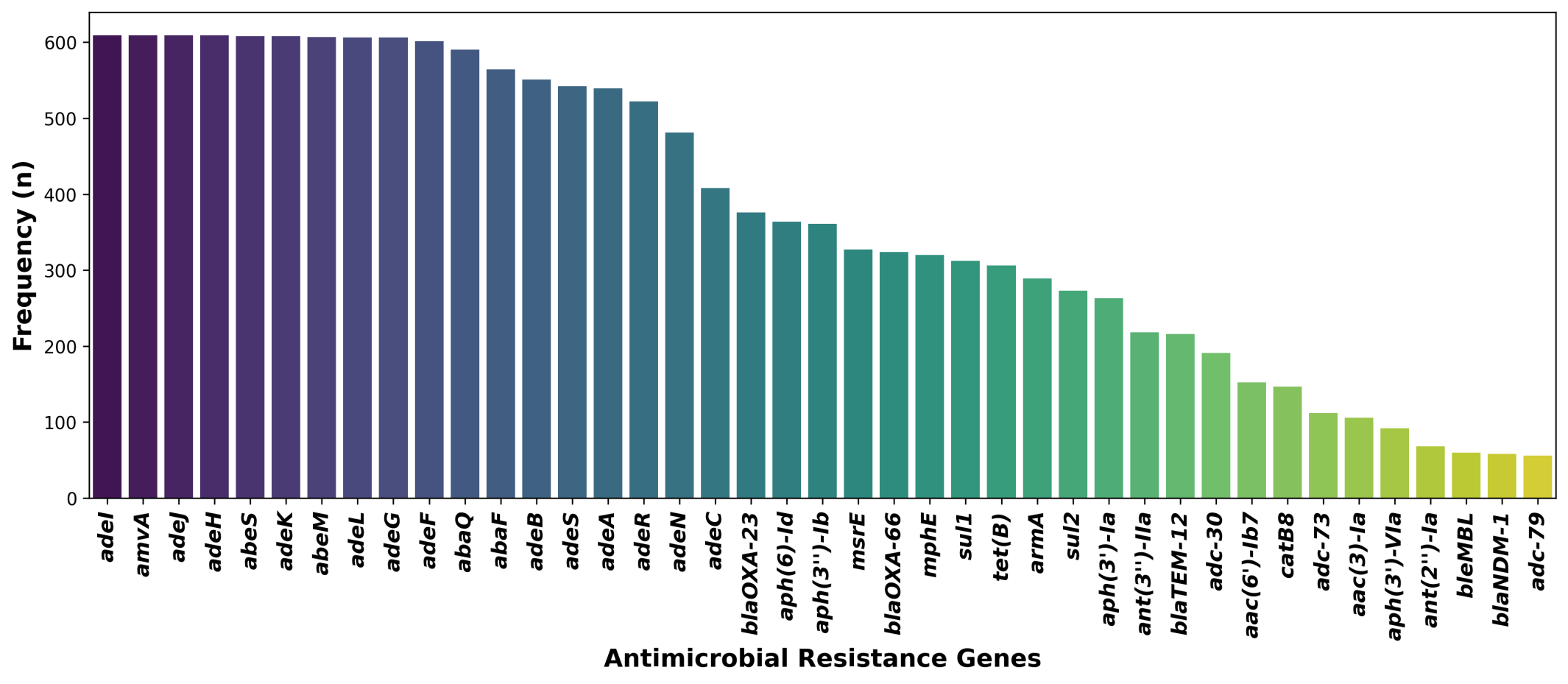


**Supplementary Fig. S4:** Bar plot representing the frequency of prevalent ARGs in the genomic dataset


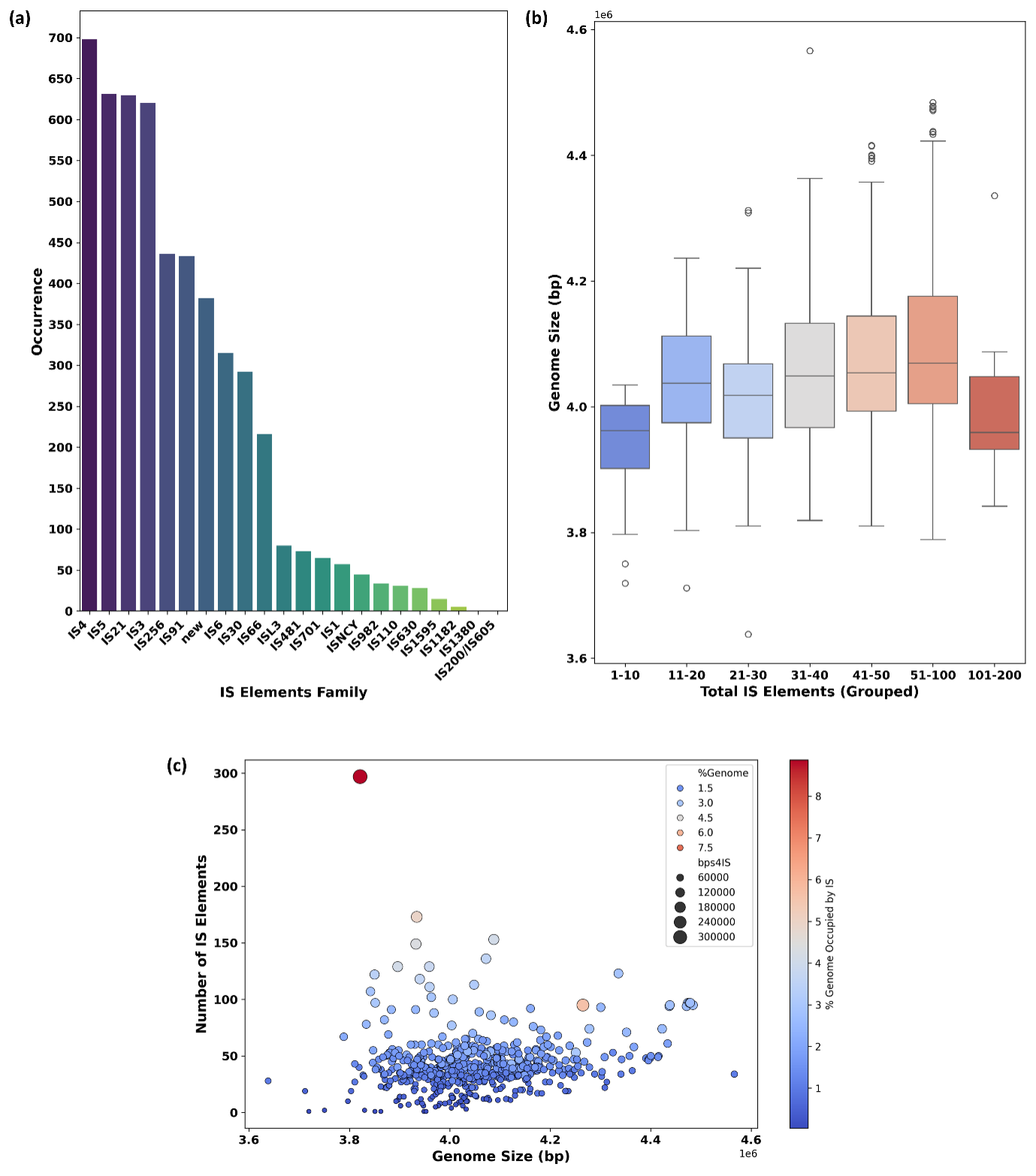


**Supplementary Fig. S5:** (a) Bar plot showing the occurrence of IS element families in the dataset (b) Box plot depicting the relationship between the total number of IS elements and genome size (c) Scatter plot illustrating the correlation between genome size and the number of IS elements


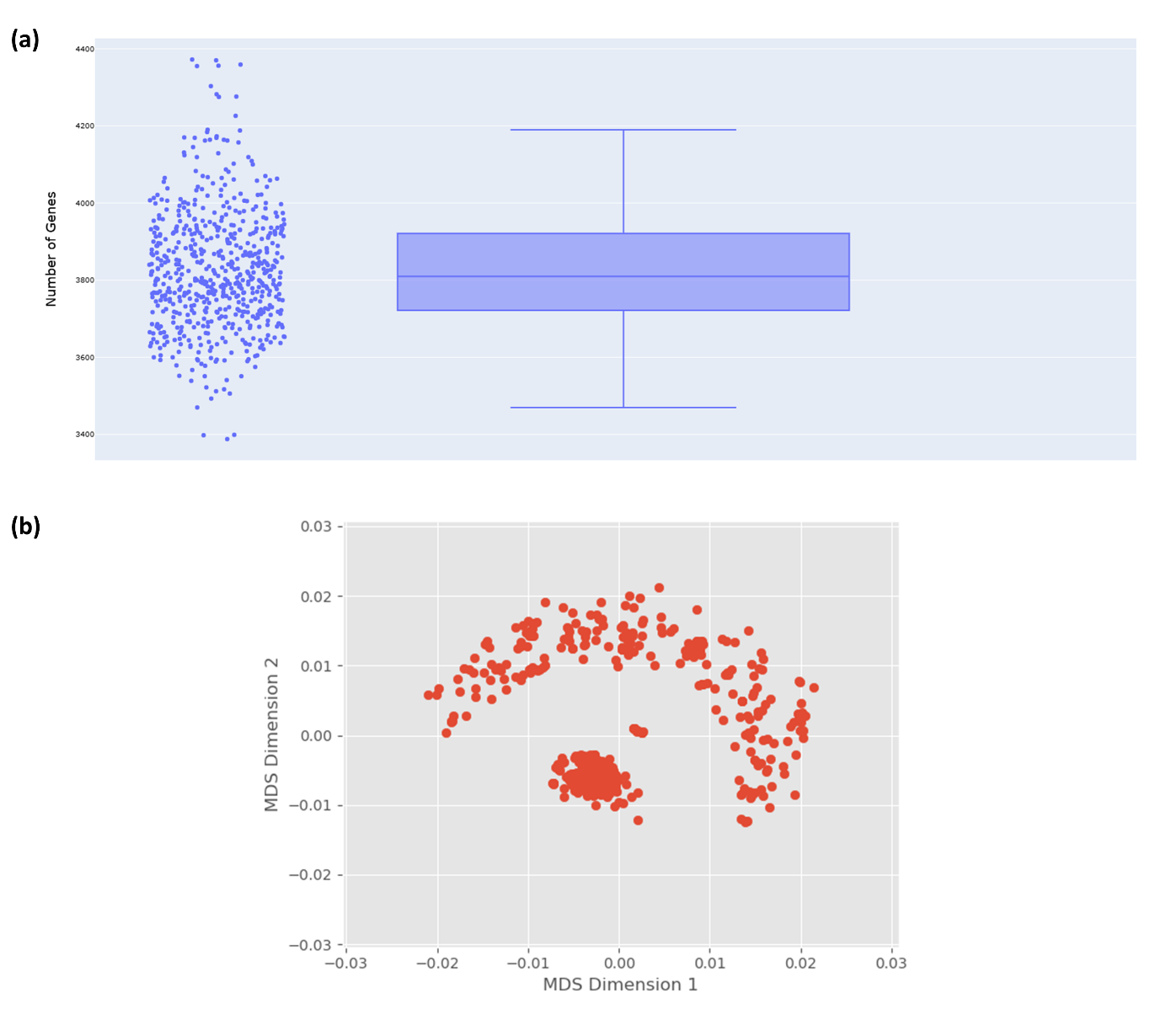


**Supplementary Fig. 6:** (a) Box plot showing the distribution of the number of genes per genome in the dataset (b) Multidimensional scaling (MDS) plot illustrating the relationships among the genomes in the dataset


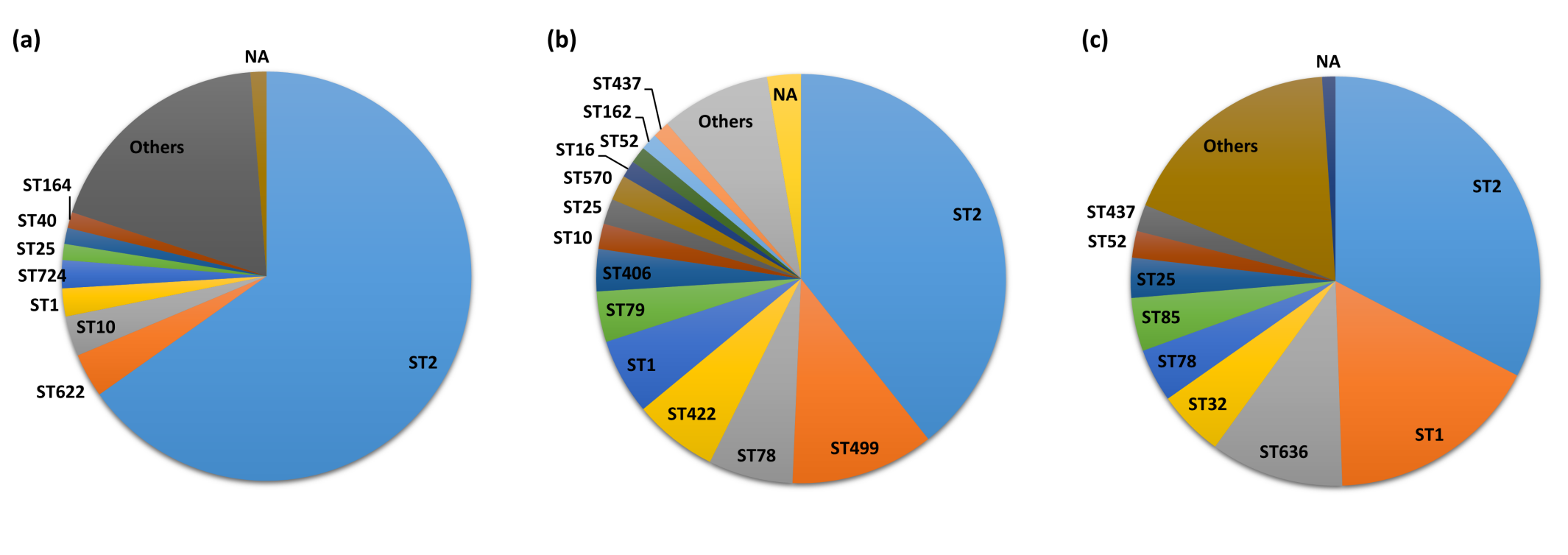


**Supplementary Fig. 7:** Geographical distribution of sequence types across (a) Asia (b) the Americas (c) Europe
